# Substitution of Glutamate Residue by Lysine in the Dimerization Domain Affects DNA Binding Ability of HapR by Inducing Structural Deformity in the DNA Binding Domain

**DOI:** 10.1371/journal.pone.0076033

**Published:** 2013-10-14

**Authors:** Richa Singh, Yogendra Singh Rathore, Naorem Santa Singh, Nagesh Peddada, Ashish, Saumya Raychaudhuri

**Affiliations:** CSIR-Institute of Microbial Technology, Molecular Biology Division, Chandigarh, India; University of Alabama at Birmingham, United States of America

## Abstract

HapR has been given the status of a high cell density master regulatory protein in *Vibrio cholerae*. Though many facts are known regarding its structural and functional aspects, much still can be learnt from natural variants of the wild type protein. This work aims at investigating the nature of functional inertness of a HapR natural variant harboring a substitution of a conserved glutamate residue at position 117 which participates in forming a salt bridge by lysine (HapR_V2G_-E^117^K). Experimental evidence presented here reveals the inability of this variant to interact with various cognate promoters by *in vitro* gel shift assay. Furthermore, the elution profiles of HapR_V2G_-E^117^K protein along with the wild type functional HapR_V2G_ in size-exclusion chromatography as well as circular dichroism spectra did not reflect any significant differences in its structure, thereby indicating the intactness of dimer in the variant protein. To gain further insight into the global shape of the proteins, small angle X-ray scattering analysis (SAXS) was performed. Intriguingly, increased radius of gyration of HapR_V2G_-E^117^K of 27.5 Å in comparison to the wild type protein from SAXS data analyses implied a significant alteration in the global shape of the dimeric HapR_V2G_-E^117^K protein. Structure reconstruction brought forth that the DNA binding domains were substantially “parted away” in this variant. Taken together, our data illustrates that substitution of the conserved glutamate residue by lysine in the dimerization domain induces separation of the two DNA binding domains from their native-like positioning without altering the dimeric status of HapR variant.

## Introduction

*Vibrio cholerae* is endowed with a remarkable capacity to sense diverse environmental settings and accordingly modulate disparate cellular events to maintain its sustainability in a given niche. The process of sensing and further coordination of cellular activities is largely dependent on a microbial social networking program known as quorum sensing. In recent past, an explosive growth in studies on quorum sensing of *Vibrio cholerae* clearly illustrates the intricacy of such complex signal transduction at the molecular level and its influence on the lifestyle of this bacterium. The intricate architecture of sensory circuit has revealed the participation of myriad of factors, namely (i) two autoinducer systems (AI-2/LuxPQ and CAI-1/CqsS), (ii) one growth phase-regulated VarS/A-CsrA/BCD unit, (iii) a small nucleoid protein, Fis and (iv) a cascade of small RNAs, plays a critical role in balancing the periodic appearance and the performance of master regulatory proteins LuxO and HapR [1]. Being the high cell density master regulatory protein, HapR is under the spotlight as it governs a constellation of disparate physiological events, thereby shaping the virulence and survival strategy of *Vibrio cholerae* [2]. Collectively, it represses biofilm development and the production of primary virulence factors, while stimulates the production of HA/protease, promotes chitin induced competence, increases resistance to protozoan grazing, enhances the survival against oxidative stress and controls the expression of gene encoding Hcp [3]. Structurally, the protein comprises of two main domains namely DNA binding domain (DBD) and dimerization domain (DD). There are a total of nine α helices where the first three helices form a putative DNA binding domain. The HTH motif comprises of helices α2 and α3. The remaining six α-helices are distributed in the C-terminal dimerization domain [4]. In a continuing effort, Zhu and coworkers have elegantly characterized additional novel direct targets of HapR and illustrated two distinct binding motifs (motif 1 and motif 2) in various target promoters. Motif 1 sequence contains dyad symmetry and a consensus AATAR′ (where R represents A or G) whereas motif 2 sequence lacks any dyad symmetry and contains a highly conserved consensus ‘TGT’ [5]. Though many crucial facts about the structural and functional aspects of wild type HapR are available, additional insights into the functionality of this molecule can further be explored by studying natural variants of the same. For instance, a natural non functional HapR variant harboring aspartate in place of glycine residue at position 39 (G^39^D; named as HapR_V2_) clearly elucidates the contributory role of glycine^39^ in DNA binding activity of HapR [6]. Further, structural analysis reveals the mutation driven shape change in the DNA binding domains, thereby explaining the loss in DNA binding ability of this HapR variant. In short, molecular analysis of HapR_V2_(G^39^D) natural variant illustrates the importance of a conserved glycine residue in the performance of HapR [6] which was not evaluated in the previous studies [4].

A flurry of research articles evidences the preponderance of natural variants of quorum sensing regulatory proteins from various strains of *Vibrio cholerae* [6]–[10]. Of these, strains having non-functional HapR variants are more frequently obtained in nature. In a recent effort, Zhu and colleagues have reported the identification of several quorum sensing defective strains of *Vibrio cholerae* harboring either a single or multiple mutations in HapR protein [10]. Though, these natural variants of HapR have been categorized as non functional based on certain phenotypic traits, further molecular analysis is required to understand the cause of their functional inertness. In this work, we have chosen to investigate the molecular basis of the functional impairment of a HapR natural variant harboring substitution of a conserved glutamate residue (E^117^) at position 117 with lysine in the dimerization domain. In literature, similar substitution where glutamate is replaced by lysine could result in mutant proteins either with a compromise in DNA binding ability or dimer stability. For example, glutamate to lysine substitution in many cases such as E^62^K and E^95^K severely affect the DNA binding ability of yeast transcription factor (TF) IIB and Oct-1 respectively [11], [12]. Similarly, analogous substitution (E^99^K) destabilizes the dimer of *Vibrio harveyi* flavin reductase FRP [13]. It should be noted that glutamate at position 99 forms a salt bridge, thereby contributing to dimer stability of *Vibrio harveyi* flavin reductase FRP [13]. With this background, we wanted to investigate the effect of replacement of a conserved salt bridge glutamate residue by lysine (E^117^K) on the stability as well as DNA binding activity of HapR_V2G_-E^117^K variant. Herein, our data reveals the intactness of dimeric status in HapR_V2G_-E^117^K variant with a compromise in DNA binding activity. To correlate the loss of function to its global shape, SAXS experiments were carried out. Data analysis and structure reconstruction revealed that though HapR_V2G_-E^117^K variant remains dimeric, its DNA binding domains are significantly open compared to the wild type functional protein. Overall, our work offers clear evidence to support a proposal that the widening of space between the DNA binding regions impairs the ability of HapR_V2G_-E^117^K to effectively bind to its cognate promoter regions.

## Materials and Methods

### Bacterial strains and media

The bacterial strains and plasmids used in this study are listed in Table 1. *Vibrio cholerae* strains were derived from a non-O1, non-O139 strain V2, serogroup O37. Strains were maintained at −70°C in Luria-Bertani (LB) medium containing 20% glycerol. *Escherichia coli* BL21 (DE3) (Novagen) was used for the over-expression of proteins. All strains were propagated at 37°C in liquid with agitation or on solid (1.5% agar) in Luria Broth unless mentioned otherwise. For protease assay, *V. cholerae* strains were grown with aeration at 37°C in tryptic soya broth without dextrose (TSB-D). When appropriate, the growth medium was supplemented with ampicillin (100 µg ml ^−1^) and chloramphenicol (17 µg ml ^−1^). All antibiotics were purchased from Sigma-Aldrich and media ingredients from Himedia and Difco.

**Table 1:** Strains and plasmids used in this study.

Strains/plasmids	Description	Source/reference
***V. cholerae*** ** strains**		
V2	Non-O1, non-O139, Sergroup O37	Ranjan K Nandy, National Institute of Cholera and Enteric Diseases (NICED),India
V2s	Non-O1, non-O139, Sergroup O37, *hapR*::pCD, Cm^r^ (17 µg ml^−1^)	Dongre *et.al*, 2011
V2_S_-C	Non-O1, non-O139, Serogroup O37, *hapR*::pCD carrying pKK177-3R1, Cm^r^ (17 µg ml ^−1^), Ap^r^(100 µg ml ^−1^)	Dongre *et.al*, 2011
V2_S_-R_V2G_	Non-O1, non-O139, Serogroup O37, *hapR*::pCD having pSV2G, Cm^r^ (17 µg ml ^−1^), Ap^r^(100 µg ml^−1^)	Dongre *et.al*, 2011
V2_S_ -R_V2G_–E^117^K	Non-O1, non-O139, Serogroup O37, *hapR*::pCD having pSV2G-E117K, Cm^r^(17 µg ml ^–1^), Ap^r^(100 µg ml ^–1^)	This study
V2_S_ –R_V2G_–FLAG	Non-O1, non-O139, Serogroup O37, *hapR*::pCD having pSV2G-FLAG, Cm^r^(17 µg ml ^–1^), Ap^r^(100 µg ml ^–1^)	This study
V2_S_ -R_V2G_–E^117^K-FLAG	Non-O1, non-O139, Serogroup O37, *hapR*::pCD having pSV2G-E117K-FLAG, Cm^r^(17 µg ml ^–1^), Ap^r^(100 µg ml ^–1^)	This study
***E.coli*** ** strains**		
Nova blue	*E. coli* K-12, *recA endA*, *lacI ^q^*, *lacY*	Novagen
BL21(DE3)	*E. coli* B, *F^–^ompT lon*, with a λ prophage carrying the T7 RNA polymerase	Novagen
**Plasmids**		
pKK177-3R1	Ap^r^	Giesla Stroz, National Institute of Health, U.S.A.
pET15b	Ap^r^, N-terminal 6His-tag expression vector	Novagen
HapR_V2_-pET15b	612 bp fragments of *hapR* (ORF) containing D^39^ (V2) was cloned into *NdeI-BamHI* site of pET15b	Dongre *et.al*, 2011
HapR _V2G_-pET15b	612 bp fragments of *hapR* (ORF) containing G^39^ (V2G) was cloned into *NdeI-BamHI* site of pET15b	Dongre *et.al*, 2011
HapR _V2G_ -E117K-pET15b	612 bp fragments of *hapR* (ORF) containing K^117^(V2G-E^117^K) was cloned into *NdeI-BamHI* site of pET15b	This study
pSV2G	612 bp fragment of functional *hapR* (ORF) cloned into SmaI- HindIII site of pKK177-3RI	This study
pSV2G-E117K	pSV2G with lysine at position 117	This study
pSV2G-FLAG	*hapR* tagged with FLAG. FLAG and *hapR* fragments were amplified from the p3XFLAG-CMV-10 Expression Vector (Sigma Aldrich) and pSV2G with primer pairs O L FLAG F/ *HindIII* FLAG R and *SmaI* HapR F/O L FLAG R, respectively. The products were purified and overlapping PCR was performed with primer pair *SmaI* HapR F/*HindIII* FLAG R. The final product was cloned into *SmaI/HindIII* sites of pKK177 3RI.	This study
pSV2G-E117K-FLAG	*hapR* (E117K) tagged with FLAG. FLAG and *hapR* fragments were amplified from the p3XFLAG-CMV- 10 Expression Vector and pSV2G-E117K with primer pairs O L FLAG F/ *HindIII* FLAG R and *SmaI* HapR F/O L FLAG R, respectively. The products were purified and overlapping PCR was performed with primer pair *SmaI* HapR F/*HindIII* FLAG R. The final product was cloned into *SmaI/HindIII* sites of pKK177-3RI.	This study

### Protease assay

Protease activity was measured using an azocasein assay as described earlier [7]. Briefly, the recombinant derivatives of *V. cholerae* strain V2s (Table 1) were grown in tryptic soya broth without dextrose (TSB-D), containing chloramphenicol (17 µg ml ^–1^) and ampicillin (100 µg ml ^–1^) accordingly, with agitation to stationary phase at 37°C. 100 µl of stationary phase culture supernatant was incubated with 100 µl azocasein (5 mg ml ^–1^ in 100 mM Tris, pH 8.0) for 1 h at 37°C. The reaction was stopped by the addition of 400 µl of 10% tricholoroacetic acid. After centrifugation, supernatant was transferred to 700 µl of 525 mM NaOH, and the optical density was determined at 442 nm. One azocasein unit was defined as the amount of enzyme producing an increase of 0.01 OD units per hr.

### Site- specific mutagenesis

Glutamate 117 to lysine (E^117^K) mutation in the functional *hapR* ORF was generated on plasmid pSV2G (Table 1) using quick change mutagenesis kit from Stratagene as per manufacturer's guidelines. The primers are listed in Table 2. Positive clones were analyzed by sequencing in their entirety to confirm the desired mutation. One of such mutant clone designated as pSV2G-E117K was further transformed into V2_S_ and the recombinant strain was designated as V2_S_-R _V2G_-E^117^K.

**Table 2:** Primers used in this study.

Primer name	Primer sequence (5′-3′)
HapR *SmaI* (cloning and sequencing primer)	TAACCCGGGATGGACGCATCAATC
HapR *HindIII* (cloning and sequencing primer)	CCCAAGCTTCTAGTTCTTATAGATAC
HapR E^117^K F (mutagenesis primer)	GGCTCAAAGTCTGGTTTAAGTGGAGTGCTTCAACC
HapR E^117^K R (mutagenesis primer)	GGTTGAAGCACTCCACTTAAACCAGACTTTGAGCC
*NdeI* HapR (cloning and sequencing)	GGGAATTGCATATGATGGACGCATCAATCGAAAAAC
*BamHI* HapR (cloning and sequencing)	CGCGGATCCCTAGTTCTTATAGATACACAG
p*vc0900* F (promoter vc0900)	TGGTTTAGTCGAGAGCTACTGCCG
p*vc0900* R (promoter vc0900)	GAGTGAAGGCCAAAGTCATTG
YF13(promoter *aphA*)	GATCGGAATTCGAATGCGCAATACTGGTTAAC
YF12(promoter *aphA*)	GATCGGGATCCGATAACGTGTGGTAATGACATG
p*hapA* F (promoter *hapA*)	GGAATTCGACTCATGGGGACTTGC
p*hapA* R (promoter *hapA*)	GGAATTCTCAGTTGCCGCTCCGGCCA
O L FLAG F	GTGTATCTATAAGAACGACTACAAAGACCATG
O L FLAG R	CATGGTCTTTGTAGTCGTTCTTATAGATACAC
HindIII FLAG R	CCCAAGCTTTTACTTGTCATCGTCATC

### Protein purification and Electrophoretic gel mobility shift assay with promoter regions of *aphA, hapA* and *vc0900*

HapR_V2G_ and HapR_V2G_-E^117^K proteins were purified by Ni^2+^-Nitrilotriacetic acid chromatography as described previously [6]. Both wild type and variant HapR were cloned into *Nde*I-*BamH*I site of the pET15b vector (Novagen) to generate N-terminal 6X His-HapR fusion proteins. All the clones were confirmed by sequencing and transformed into *E. coli* BL21 (DE3). After induction with 0.4 mM isopropyl 1-thio-β-D-galactopyranoside (IPTG), HapR proteins were purified through Qiagen Ni^2+^-nitrilotriacetic acid columns and subjected to overnight dialysis in a solution of buffer A containing 10 mM Tris-HCl, pH 7.9, 300 mM KCl, 0.1 mM EDTA. Gel mobility shift assay was done essentially as described earlier [6]. Briefly, three fragments of 399, 665 and 467 bp corresponding to promoter regions of *aphA*, *hapA* and *vc0900* respectively, were amplified with primer pairs as listed in Table 2. The fragments were gel-purified and end labeled with [γ^32^–P] dATP using T4 polynucleotide kinase (New England Biolabs). The binding reaction was carried out with 4 ng of labeled fragment in 10 mM Tris-HCl, pH 7.9, 1 mM EDTA, 1 mM DTT, 60 mM KCl, 10% glycerol, 5 µg BSA and 1 µg poly (dI-dC) in a 20 µl reaction volume for 20 min at 26°C. The reaction mixture was applied to a 5.5% polyacrylamide gel and subjected to electrophoresis in 1X Tris-Acetate-EDTA, pH 8.5, at 4°C. The gel was dried and autoradiographed to examine the shift of the band.

### Circular Dichroism (CD) measurement

Both HapR_V2G_ and HapR_V2G_-E^117^K were examined by CD using a Jasco J-810 spectropolarimeter. Measurements in the far ultraviolet region (250–200 nm) were performed on protein solutions (0.2 mg/ml) employing a cell with path length of 0.1 cm at 25°C. The mean residue ellipticity, [θ], was calculated using a mean residue molecular mass for both the proteins. Each spectrum reported is an average of 10 scans.

### Molecular weight determination

**Figure 1:**
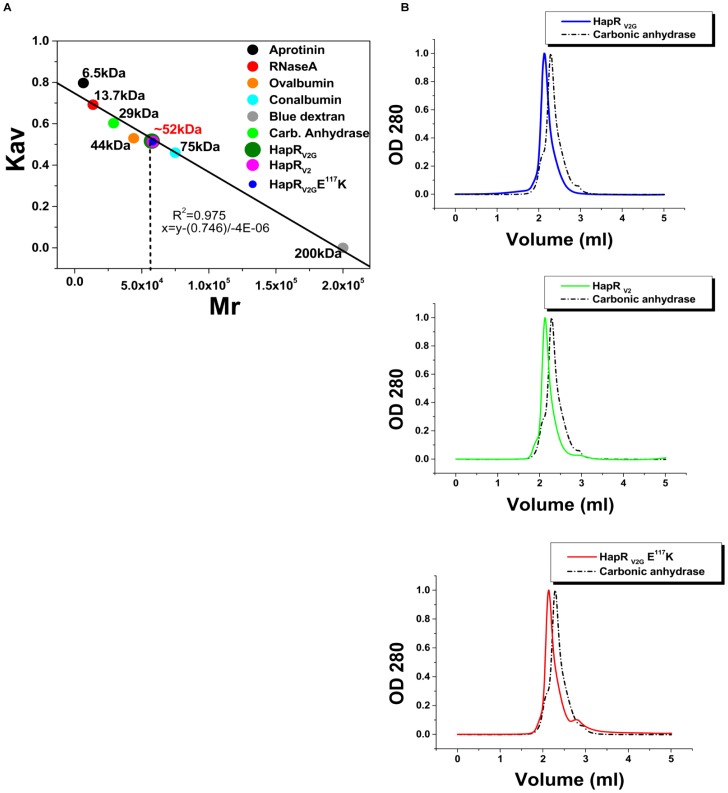
Analytical molecular sieve chromatography to determine the molecular weight of the functional (HapR_V2G_) and non-functional proteins (HapR_V2_ and HapR_V2G_-E^117^K). (A) A calibration curve of column was obtained by plotting the *K*av of the protein standards against the *M*r of the standards. Six GE-Healthcare gel filtration molecular mass markers were used: Blue dextran (200 kDa), Conalbumin (75 kDa), Ovalbumin (44 kDa), Carbonic anhydrase (29 kDa), Ribonulcease A (13.7 kDa) and Aprotinin (6.5 kDa). Each marker along with its *M*r is represented by a unique colour. *K*av values for all the proteins obtained from the Eq. 1 were fitted to calibration curve to estimate the molecular weight of proteins (∼52 kDa). (B) The elution profiles of the functional (HapR_V2G_) and non-functional proteins (HapR_V2_ and HapR_V2G_-E^117^K). Column was equilibrated in 10 mM Tris, pH7.9, 100 mM KCl, and 0.1 mM EDTA. Elution profile of Carbonic anhydrase (29 kDa) is shown in dotted line for comparison.

HapR_V2G_, HapR_V2_ and HapR_V2G_-E^117^K were dialyzed overnight in buffer A. All proteins were subjected to molecular sieve chromatography using a Superdex 200 5/150 GL analytical column coupled to an “AKTA-Purifier” chromatography system (GE-Healthcare). The molecular weight (*M*r) of all the proteins were evaluated according to a calibration curve generated with the gel filtration of molecular mass markers (GE-Healthcare) including blue dextran (200 kDa), conalbumin (75 kDa), ovalbumin (44 kDa), carbonic anhydrase (29 kDa), ribonulcease A (13.7 kDa) and aprotinin (6.5 kDa) (Figure 1A). These standards were loaded independently at the concentrations recommended by GE-Healthcare. The elution volume of blue dextran was used to determine the void volume (*Vo* = 1.215 ml) and the total volume (*Vt* = 3 ml) was provided by the product instruction manual. The peak elution volumes (*Ve*) were calculated from the chromatogram. *K*av were calculated using the following equation:
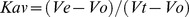
(1)


The calibration curve was determined by plotting the *K*av of the protein standards against the *M*r of the standards. Elution volumes were determined by monitoring the absorbance at 280 nm. All proteins were eluted at the same volume. Kav values for all the proteins were calculated based on elution volumes and fitted to a calibration curve to estimate the size of the proteins.

### Sodium dodecyl sulfate-polyacrylamide gel electrophoresis (SDS- PAGE) and western blot analysis

Western blot analysis was done to check the expression of HapR. Briefly, FLAG- tagged recombinant derivatives of *V. cholerae* strain V2s were grown overnight in TSB-D medium with agitation. Protein samples were prepared from equal amount of bacterial cells and separated on 13.5% gel by SDS-PAGE. For immunoblotting, the proteins were transferred to Immobilon-P membrane (Millipore) at 75 mA for 1 h. Membrane was subsequently blocked in PBS-T with 5% skim milk at 37°C for 2 h and then shifted to 4°C for overnight with shaking. Blot was then washed in PBS-T five times for 10 min each, incubated in monoclonal HRP- conjugated Anti-flag (Sigma Aldrich) at a dilution of 1:5000 in PBS-T with 2% skim milk for 1 h and again washed in PBS-T five times for 10 min each. Proteins were visualized using the Luminata Forte western HRP substrate (Millipore). Molecular masses were calculated with reference to SDS-PAGE molecular mass standards (Broad range) from NAX-GEN ALPHA PS ladder from Genetix biotech.

### Small Angle X-ray Scattering Data Acquisition, Analysis and Structure Reconstruction

All SAXS intensity profiles were acquired at the X9 beam line (National Synchrotron Light Source, Brookhaven National Laboratory). Scattered X-rays were recorded as images on Pilatus detectors, corrected for slit smearing, circularly averaged about the beam centre, and the contribution from buffer components were subtracted using Python script based programs written by Dr. Lin Yang (X9 Beam line, NSLS). For each experiment, 120 µl of protein solutions (HapR_V2G_ and HapR_V2G_-E^117^K) and their matched buffers from FPLC were exposed to X-rays in a quartz capillary flow cell. Using programmable liquid handling robotics at X9 beam line, the collection of entire SAXS data described in this study were carried out in triplicate and averaged during processing. To calibrate the beam intensity at zero angles and estimate the actual concentration of protein samples, the SAXS datasets were collected under identical conditions on a series of hen egg white lysozyme (in NaOAc buffer containing 150 mM NaCl, pH 3.8) and recombinant gelsolin (in Tris-EGTA, buffer pH 8) with their predetermined concentrations. Image processing provided the scattering intensity (I) as a function of momentum transfer vector, Q (Q = [4πsinθ]/λ), where 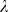 and 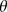 are the wavelength of X-ray and the scattering angle, respectively. Kratky plots (I (Q) Q^2^ *vs*. Q) were generated to interpret globular scattering nature of the wild type and variant protein in solution. The Guinier approximations of the low Q region were carried out using PRIMUS software [14] presuming globular and rod-like scattering shape which provided the radius of gyration (R_G_) and radius of cross-section (R_C_) of the predominant shape of protein molecules. Using the relationship between R_G_ and R_C_ [Eq. 2], we estimated the linear dimension of the molecules with following equation:
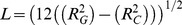
(2)


Further, indirect Fourier transformation of the SAXS data (Q range: 0.008–0.25 Å^-1^) was performed using GNOM45 program [15] to obtain probability of finding various pairwise vectors arising from the scattering shape of the protein molecules. During P(r) curve estimation, the probability of finding a pairwise vector of length equal to 0 Å and equal to the maximum linear dimension (D_max_) of the molecule was considered to be zero. This analysis also provided an estimate of the R_G_ and scattering intensity at zero scattering angles, I_0_. To visualize the three dimensional shapes of wild type and the variant HapR protein, ten independent models of each were generated using the DAMMINIQ program and the respective SAXS I(Q) profile as reference [16]. No symmetry or shape bias was employed during each shape reconstruction. For each protein, the independent structural models were averaged using DAMAVER suite of programs [17] which resulted in a structural model capable of best-representing the solution shape of the proteins in solution. The SAXS data based dummy model and the available crystal structure of HapR (PDB ID: 2PBX) were overlaid by aligning inertial axes of the models using SUPCOMB20 program [18]. The open source VMD version 1.9.1 and Chimera 1.6.1 were used for graphical analysis and figure generation.

## Results and Discussion

### Molecular weight and secondary structure determination of non-functional HapR_V2G_-E^117^K

**Figure 2:**
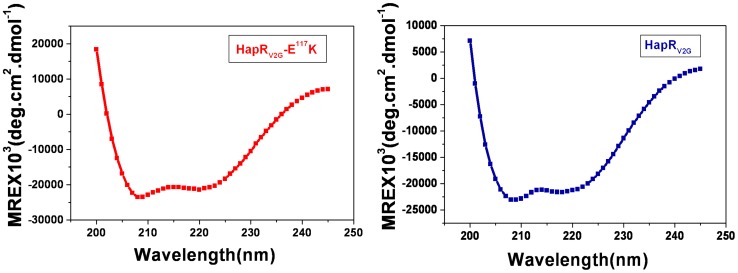
Circular dichroism spectra for functional HapR(HapR_V2G_) and non-functional HapR (HapR_V2G_-E^117^K). Far-UV CD spectra of proteins were obtained between wavelengths 250 and 200 nm. Mean residual ellipticity (*MRE*) was calculated and plotted against the wavelength.

Like other members of TetR family proteins, HapR also acts as a dimer [4]. Analysis of the dimerization domain and interface reveals the significance of critical residues in the formation and stabilization of dimer. One such residue glutamate at position 117 (E^117^) contributes to the stability of the HapR dimer by forming a salt bridge with arginine residue at position 123 (R^123^) of the other monomer [4]. There is a preponderance of data elucidating the crucial role of salt bridge in stabilizing protein structure. For example, substitution of a glutamate residue at position 99 with lysine (E^99^K) in case of *Vibrio harveyi* flavin reductase FRP completely abolishes the dimer formation. It should be noted that E^99^ makes salt bridge with R^133^ and R^225^ of the other monomer in FRP [13]. In our study, we were driven by a desire to understand the molecular basis of the non-functionality of previously identified HapR variant harboring substitution of a conserved glutamate residue at position 117 (E^117^) with lysine. As described in the preceding section, E^117^ of a functional wild type HapR participates in the formation of salt bridge, it is therefore conceivable that substitution of this residue by lysine in non-functional HapR variant (HapR_V2G_-E^117^K) may alter dimer stability, thereby affecting its functionality. To ascertain, we estimated the molecular weight of proteins by size-exclusion chromatography. The *K*av values of all the proteins were obtained using Eq. 1 and put in equation x = y−(0.746)/-4E-06 to calculate their respective molecular weights (Figure 1A). In addition to the wild type functional protein as control, we also kept a previously identified non functional HapR variant (HapR_V2_) that is defective in DNA binding but forms a stable dimer [6] as a second control in gel filtration chromatography experiment (Figure 1B). Intriguingly, no significant difference was observed in the gel filtration elution profiles of the purified His-tagged HapR functional (HapR_V2G_) protein, DNA binding defective variant (HapR_V2_) and dimerization domain variant (HapR_V2G_-E^117^K). We also carried out CD data analysis to examine any alteration in the secondary structure (Figure 2). K2D2 analysis of the CD data (in the range of 250–200 nm) suggested that only a minor loss of α helical content (<4%) occurred in case of HapR_V2G_-E^117^K. Taken together, our results indicate that the hydrodynamic property of HapR_V2G_-E^117^K variant is similar to that seen for the dimeric wild type HapR_V2G_ suggesting the intactness of dimeric status of HapR_V2G_-E^117^K variant.

### Evaluate DNA binding ability of variant HapR_V2G_-E^117^K

**Figure 3:**
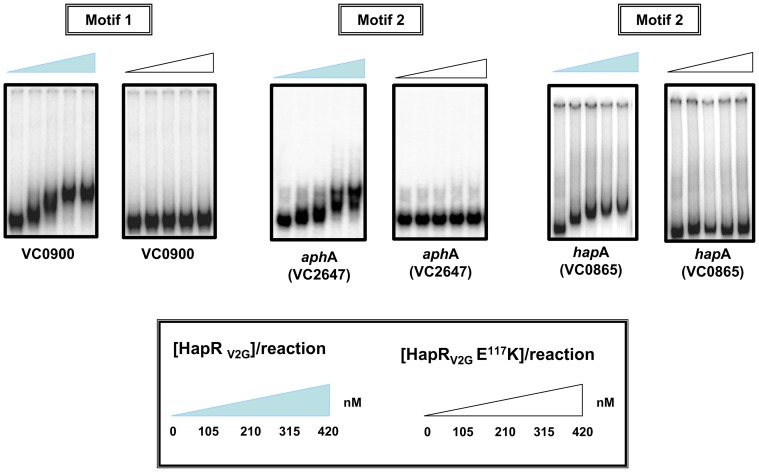
Gel shift experiments. Electrophoretic mobility shift assay of purified HapR_V2G_ and HapR_V2G_-E^117^K were carried out with ^32^P-labeled promoter regions of *vc0900*, *aphA*, and *hapA*. *Filled* and *open wedges* represent HapR_V2G_ and HapR_V2G_-E^117^K proteins respectively.

HapR justifies its role as a master regulator by interacting with a range of cognate promoter sequences. To assess the DNA binding ability of HapR_V2G_-E^117^K, a gel shift assay was employed with *hapA*, *aphA* and *vc0900* promoter regions. It should be noted that the promoter region of *vc0900* contains motif 1 binding site while the promoter regions of *hapA* and *aphA* contain motif 2 binding site. Unlike its wild type functional counterpart HapR_V2G_, HapR_V2G_-E^117^K fail to bind to any of these promoter regions, thereby indicating a compromise in DNA binding ability (Figure 3). Combining gel filtration and gel retardation results, we conclude that substitution of a salt bridge glutamate residue with lysine in the dimerization domain abrogates DNA binding activity without affecting dimeric status of HapR variant.

### Functional examination and stability of variant HapR_V2G_-E^117^K under in vivo condition

**Figure 4:**
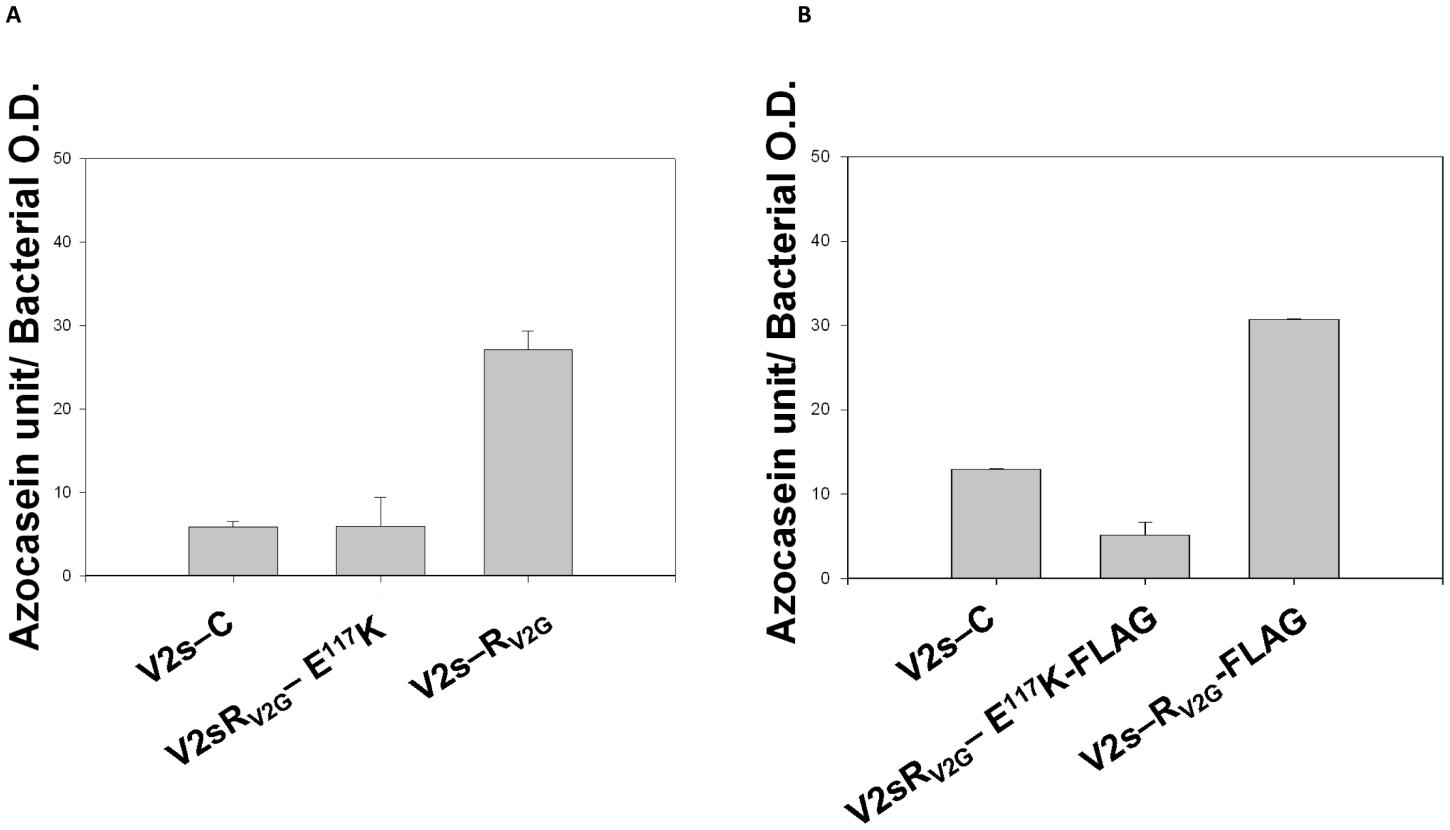
Protease activity. (A) The recombinant derivatives of V2_S_ were analyzed for protease production in the cell-free culture supernatants. The indicated strains were grown in TSB-D for 12 h at 37°C (200 rpm). Protease activity was assayed through digestion of azocasein in triplicate. (B) FLAG inserted recombinant constructs of V2_S_ were also analyzed for protease production to confirm that there is no functional alteration upon FLAG insertion in any HapR protein. Enzyme activities are the mean of three independent cultures. S.D. is indicated with *error bars*.

**Figure 5:**
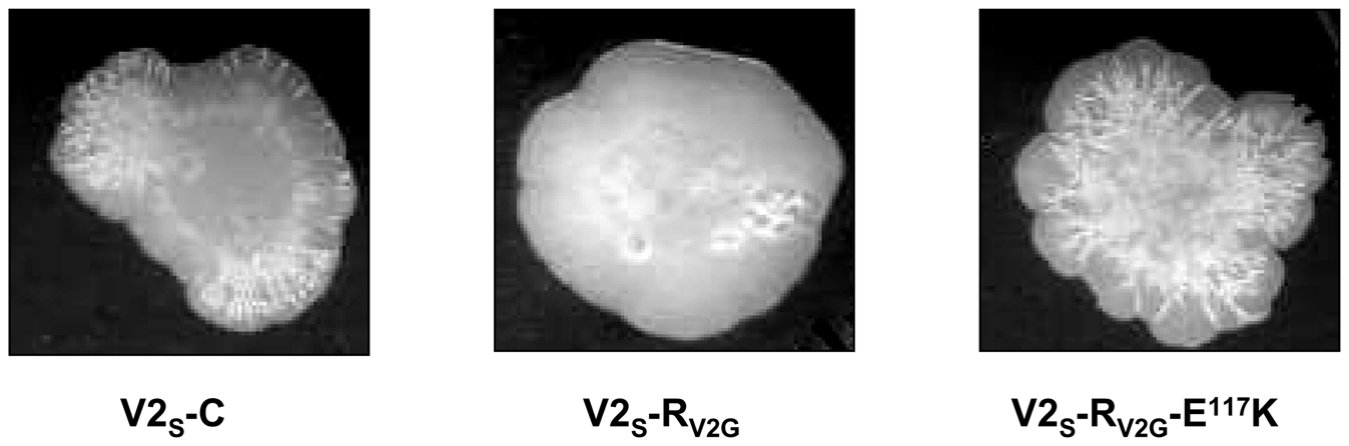
Rugosity pattern. Equal number of cells was spotted for indicated strains on LB agar supplemented with ampicillin (100 µg ml-1) and chloramphenicol (17 µg ml-1). Plates were incubated at 37°C and photographed after 48 h.

From *in vitro* gel shift assay, inability of the HapR variant (HapR_V2G_-E^117^K) to shift promoter region of gene encoding HA/protease (*hapA*) is evident. It is therefore expected that this variant will remain non functional to stimulate protease production in a protease negative strain of *Vibrio cholerae*. To ascertain its *in-vivo* functionality, recombinant constructs of *hapR_V2G_* (pSV2G, wild type functional HapR control) and *hapR-E^117^K* (pSV2G-E117K) were transformed into a protease negative *Vibrio cholerae* strain V2_S_ (*hapR* disrupted strain of V2, Table 1) to generate strains V2_S_-R_V2G_ and V2_S_-R_V2G_-E^117^K. In contrast to V2_S_-R_V2G_, V2_S_-R_V2G_-E^117^K remains protease negative (Figure 4A). The protease data recapitulates *in vitro* gel shift data with promoter *hapA* where protease production is directly proportional to the functional level of HapR. In addition to the protease production, we have also checked the rugosity pattern of V2_S_-strains harboring HapR proteins. It has been observed that strain having non-functional HapR develops rugosity (Figure 5). This data is in agreement with that of Wang *et al*, who demonstrated rugosity in the strain harboring such mutant HapR protein[10].

**Figure 6:**
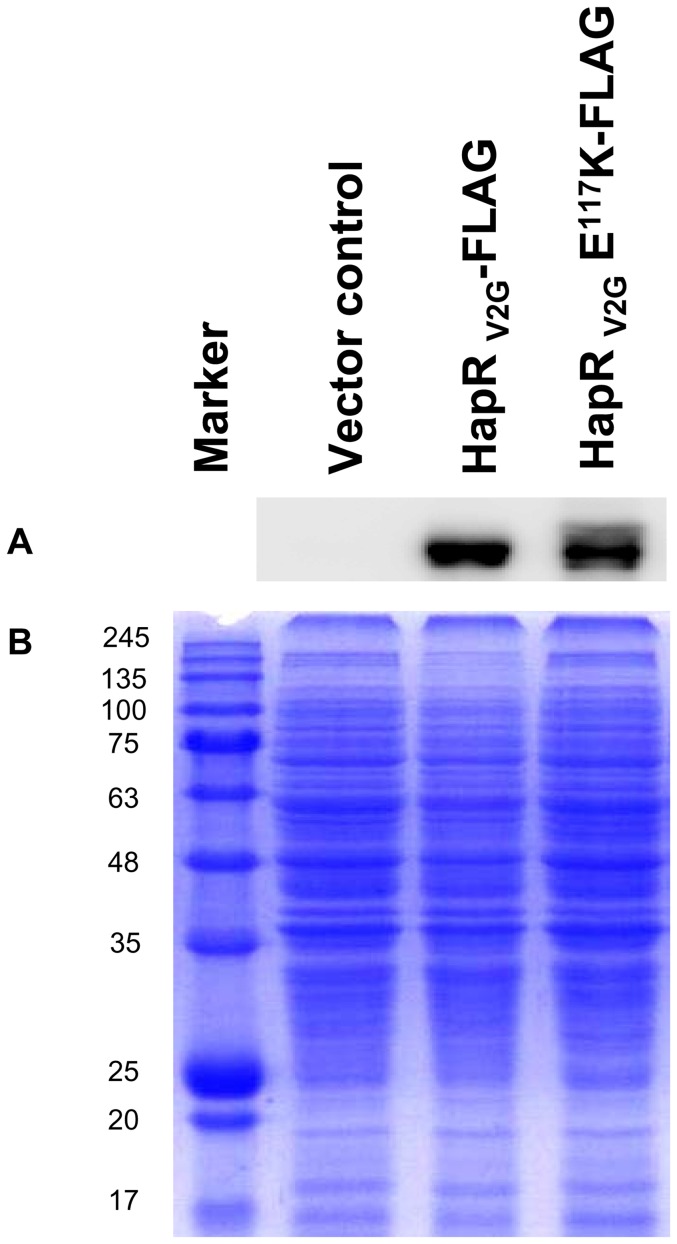
Sodium dodecyl sulfate-polyacrylamide gel electrophoresis (SDS-PAGE) and Western blot analysis of HapR production. (A) A 12-h grown culture of indicated strains were grown with agitation. Protein samples were prepared from equal number of bacterial cells, analyzed on 13.5% SDS polyacrylamide gel electrophoresis (SDS-PAGE) and subsequently transferred to Immobilon-P membrane. FLAG-tagged HapR proteins were detected using monoclonal HRP-conjugated anti-FLAG antibody. Molecular masses were calculated with reference to SDS-PAGE molecular mass standards. (B) Coomassie-brilliant-blue-stained gel is presented as loading control for western blot experiment. Protein marker sizes are shown to the left (in kDa).

It has been documented that substitution of an amino acid often results in an increased susceptibility of mutants to proteases, if critical for global stability of the folded protein [19]. It is thus possible that substitution of glutamate by lysine may affect the *in vivo* stability of HapR_V2G_-E^117^K variant. To investigate the stability, FLAG epitope was inserted in the C-terminal domain of wild type HapR_V2G_ and variant protein (Table 1). The recombinant constructs were transformed into a protease negative *Vibrio cholerae* strains V2_S_ (*hapR* disrupted strain of V2, Table 1) to generate strains V2_S_-R_V2G_-FLAG and V2_S_-R_V2G_-E^117^K-FLAG. The recombinant strains were then subjected to western blot analysis to examine the stability of each protein. Our western blot analysis clearly indicated the intactness of lysine (K^117^) variant along with wild type HapR protein (Figure 6A). The protease assay with FLAG containing recombinant constructs further confirmed no functional alteration upon FLAG insertion (Figure 4B). Until now results support that substitution of a salt bridge contact residue glutamate by lysine abrogates DNA binding ability without affecting stability and dimeric status of HapR_V2G_-E^117^K variant.

### Structural deformity of HapR_V2G_E^117^K variant as evidenced by SAXS data analysis and structure restoration

**Figure 7:**
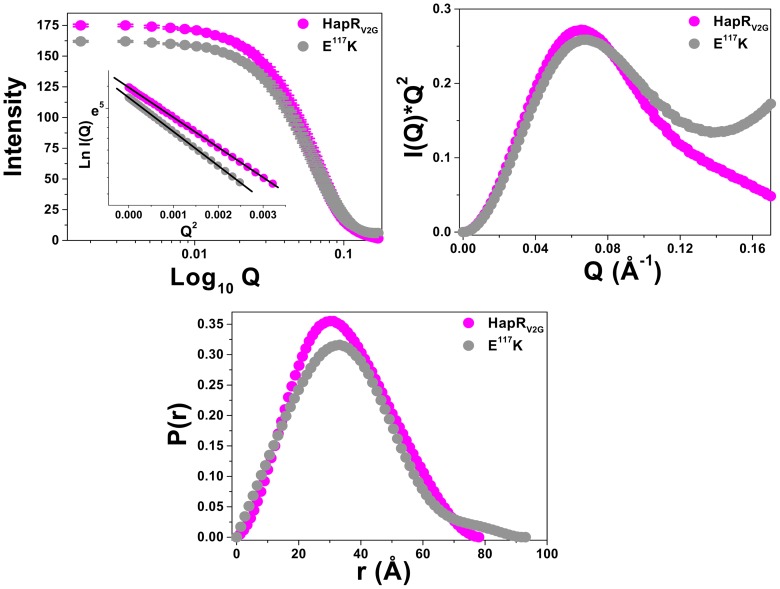
SAXS data analysis from the samples of HapR_V2G_ and HapR_V2G_-E^117^K. (A) SAXS I(Q) profiles are plotted *versus* Q for both the samples (HapR_V2G_, magenta; HapR_V2G_-E^117^K, gray). Inset shows the linear region of the Guinier analysis done presuming globular nature of the protein molecules in solution. (B) Kratky analysis from individual SAXS data sets confirmed the globular nature of proteins in solution. (C) Real space information of the predominant scattering species computed by indirect Fourier transformation are plotted between P(r) and R.

In the preceding section, our characterization of hydrodynamic, secondary structural and stability analysis of the proteins ruled out a grossly misfolded shape of HapR dimerization domain HapR_V2G_-E^117^K variant, but lacked in explaining the loss of DNA binding function. In this scenario, high resolution structural analysis may shed further light on the global architecture, thereby enabling us to understand the loss of DNA binding activity of these dimerization domain variant proteins. To further delve into the structural details, SAXS analysis was performed as described previously [6]. SAXS scattering data lacking an upturned profile in low Q region assured lack of aggregation in samples during data collection (Figure 7A). Considering globular scattering nature of the protein, Guinier analysis indicated that both wild type and variant HapR adopted a monodisperse profile in solution. Steeper slope of the linear fit for the HapR_V2G_-E^117^K variant protein (compared to wild type) indicated a bigger R_G_ for the variant (Figure 7A inset). In fact, the R_G_ values were 23.4 Å and 27.1 Å for the HapR_V2G_ (functional) and HapR_V2G_-E^117^K protein, respectively (Table 3). Presuming rod-like scattering shape for the protein molecules provided the R_C_ values for the two proteins to be 10.1 Å and 8.3 Å. Using the Eq. 2, the L values for wild type and HapR variant were estimated to be 73 Å and 89 Å, respectively. Interestingly, peak-like Kratky plot of the wild type protein confirmed a globular scattering nature of HapR_V2G_ molecules in solution, a wider peak with I(Q)*Q^2^ not dampening to zero as a function of increasing Q for the mutant suggested relatively higher inherent disorder in the HapR_V2G_-E^117^K protein molecules (Figure 7B). Increased dimensions of the variant protein were also seen from the indirect Fourier transformation of the SAXS datasets (Figure 7C). The wild type functional protein HapR_V2G_ showed P(r) curve with D_max_ and R_G_ about 78 Å and 23.9 Å, respectively [6]. At the same time, the P(r) analysis for HapR_V2G_-E^117^K protein provided D_max_ and R_G_ close to 93 Å and 27.5 Å, respectively. The computed P(r) profile for the HapR_V2G_-E^117^K variant suggested an extended curve seen for shapes with tail-like features.

**Table 3:** Structural parameters of the wild type and variant HapR proteins as deduced from their SAXS data analysis.

Proteins	Mass(KDa)	Guinier Analysis			Indirect Fourier Transformation			Actual Conc. (mg/ml)
		R_G_(Å)	R_C_(Å)	L(Å)	D_max_ (Å)	R_G_ (Å)	I_0_	
***Samples with predetermined concentrations***								
Lysozyme	14.2	14.1	7.7	41	44	14.2	20	1*
EGTA Gelsolin	82	30.4	16.4	88	100	30.1	115	1*
***HapR Proteins(Dimer)***								
HapR_V2G_	46	¶23.4±0.3	10.1	73	78	23.9±0.2	175	2.7
HapR_V2G_E^117^K	46	27.1±0.1	8.3	89	93	27.5±0.1	162	2.5

* The I_0_ values were estimated from predetermined dilution series of lysozyme (four samples in the range of 0.7–3.2 mg/ml) and recombinant plasma gelsolin (four samples in the range of 0.5–2.1 mg/ml)

¶ It is important to mention here that an error remained in our previous publication (Dongre *et.al*, 2011) during description of results from Guinier analysis: on page 15049: left column line 1 and 2 “HapR_V2_ and HapR_V2G_ provided an R_G_ of” should be read as “HapR_V2G_ and HapR_V2_ provided an R_G_ of”.

**Figure 8:**
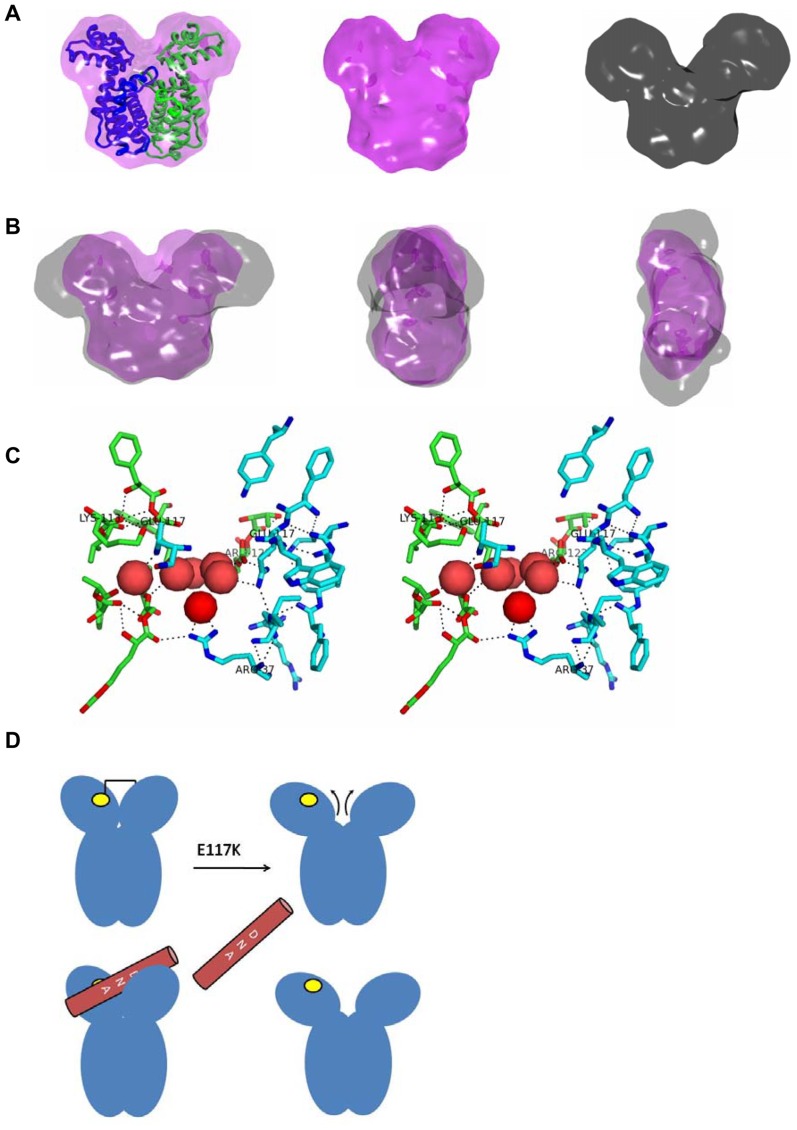
SAXS data based volume maps of HapR_V2G_ and HapR_V2G_-E^117^K. (A) Averaged model of HapR_V2G_ protein (*left*, magenta) computed from SAXS scattering data is superimposed with its respective crystal structure (PDB ID code 2PBX, chain A and B in blue and green ribbons respectively). The individual SAXS envelope profiles of functional HapR_V2G_ (*middle*, magenta) and non-functional HapR_V2G_-E^117^K variant (*right*, gray) are also presented here. (B) Superimposition of SAXS based models from both the proteins are displayed to highlight the conformational changes occurred in HapR_V2G_-E^117^K variant. For better clarity, front (*left*), side (*middle*) and top (*right*) views are shown respectively. (C) Stereo view highlighting the network of polar interactions around E^117^ in the two chains of HapR dimer (PDB ID: 2PBX). The chains are green and cyan colored in stick representation, while the oxygen and nitrogen atoms are shown in red and blue, respectively. The water molecules resolved in the crystal structure are shown in red cpk format. (D) Schematic summarizing our results that the HapR_V2G_-E^117^K mutation induces a parting in the two domains spatially optimized for DNA binding (yellow circle represents the possible docking site of DNA; one on viewable face of the figure and one behind).

To gain insight into the shape of the two proteins, *ab initio* modeling approaches were followed using dummy residues. The model solved for the predominant solution shape of HapR_V2G_ agreed well with the crystal structure of the HapR protein (PDB ID: 2PBX), as published earlier (Figure 8A). In contrast, the model solved for HapR_V2G_-E^117^K variant protein revealed a much more open structure, particularly the DNA binding domains (comparing with the HapR_V2G_). Overlay of the two models confirmed that while the shape profile of the dimerization domain of the variant remained similar to wild type protein, the DNA binding domains in mutant somewhat “fell-apart”. Moreover, this deviation in the global shape of the mutant occurred only in dimension (please see the side-views in Figure 8B). To realize how this single point mutation is causing such a deleterious effect, we analyzed the polar interactions surrounding E^117^ in the crystal structure of the HapR_V2G_ (PDB ID: 2PBX) (Figure 8C). It brought forth that alongside interacting with R^123^, HapR_V2G_-E^117^K indirectly positions other residues in vicinity *viz*. K^117^ and some water molecules. A mutation to lysine, not only reverses the local electrostatic surface potential which would alter the intricate network of interactions resolved for the native protein, but also the extended length of lysine side-chain compared to that of glutamate would find it difficult to accommodate in the groove formed between the upper portion of the dimeric interface. Replacement of two glutamates in the dimer (in the zone highlighted in Figure 8C) by two lysines would cause a substantial remodeling of the local topography. Very likely, the adjustments are done by parting away of the upper portion (since lower part of molecule is engaged in dimerization). A culmination of local changes eventually results in eventual separation of the upper domains, a geometry which has evolved for binding different double stranded DNA (Figure 8D).

## Conclusions

**Figure 9:**
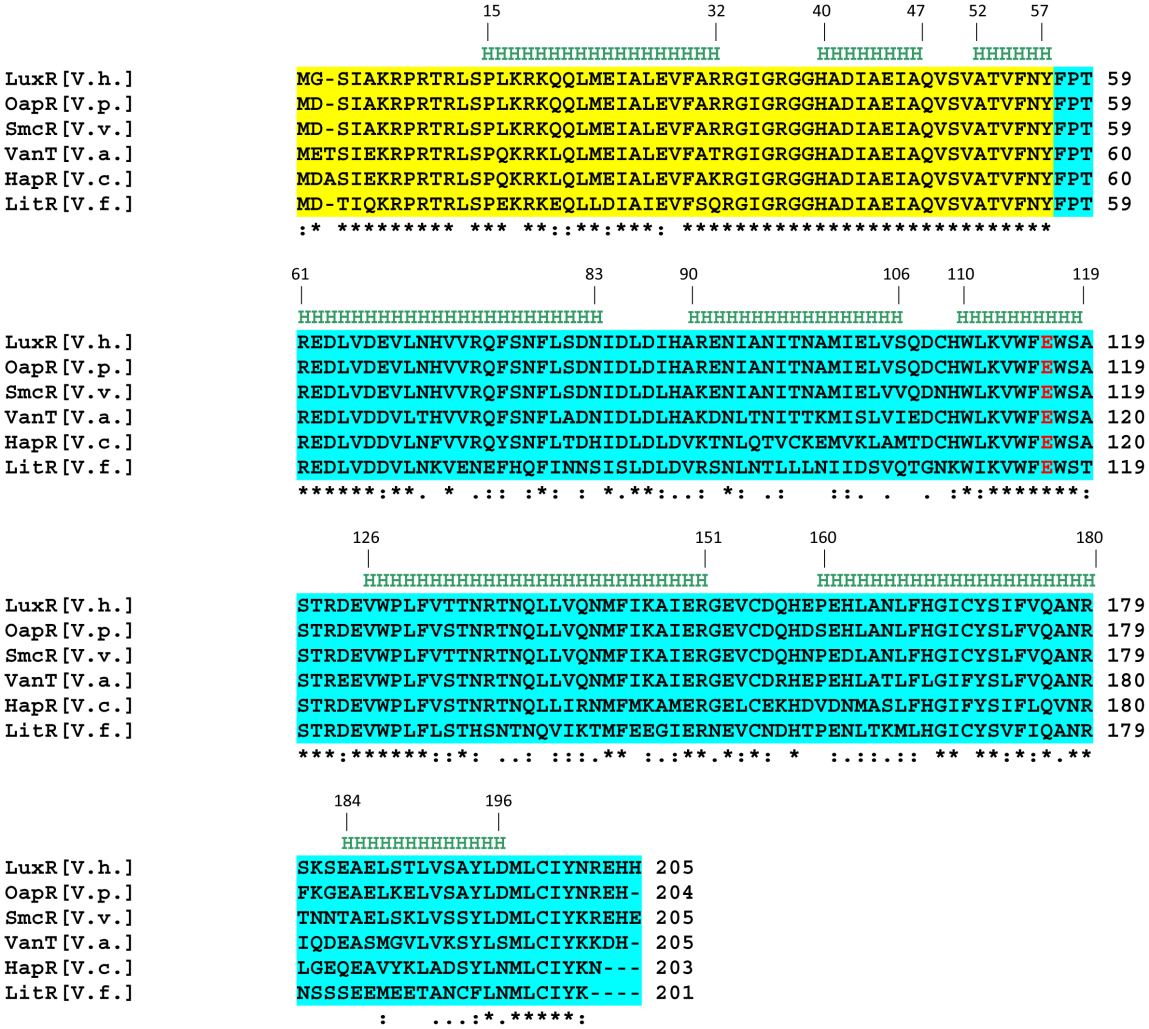
Multiple sequence alignment of HapR homologues in *Vibrio* species. Multiple sequence alignment of the deduced amino acid sequences of HapR homologues, from different *Vibrio species*. The regions shadowed in yellow and turquoise color represents the DNA binding domain and dimerization domain respectively. The conserved glutamate 117 residue in different HapR homologues is highlighted in red. Abbreviations are as follows: [V. h.] -*Vibrio harveyi*; [V.p.] –*Vibrio parahaemolyticus*; [V.v.] -*Vibrio vulnificus*; [V.a.] -*Vibrio anguillarum*; [V.c.] -*Vibrio cholerae* (C6706); [V.f.] -*Vibrio fischeri*.

There is a growing body of evidence that elucidates how substitution of a single amino acid could affect the functionality of a DNA binding protein by altering its DNA binding activity, dimeric status and overall stability by increasing susceptibility to proteases [6], [11], [13], [19]–[22]. Keeping this view in mind, we wanted to examine the underlying cause of non-functionality of a natural HapR variant designated as HapR_V2G_-E^117^K where a salt bridge glutamate residue is replaced by lysine moiety. It has been documented that similar substitution where glutamate is replaced by lysine could result in mutant proteins either with a compromise in the DNA binding ability or dimer stability [11]–[13]. A combined *in vivo* and *in vitro* analysis helped us to establish that HapR_V2G_-E^117^K variant protein forms a stable dimer with a compromise in its DNA-binding activity. As evidences suggest salt bridges not only maintain the proper orientation as well as stability of dimer interface, but also controls the wedge angle between DNA-binding domain and the dimerization domain, thereby mediating interaction between the protein molecules and their cognate DNA sequences [23], [24], [25]. In our case, replacement of a salt bridge contact residue glutamate by lysine neither affects the dimer stability nor overall protein stability, but it introduces a deformity in the DNA binding domain as revealed by SAXS analysis, thereby offering a plausible explanation of lack of DNA binding activity and overall cellular performance of HapR_V2G_-E^117^K variant. Based on our previous work and present observation, it is seemingly apparent that HapR adopts a particular “Y” shape that facilitates its interaction with cognate promoters ([6]; Figure 8). Any deviations from its typical “Y” shape either too close [6] or too widen (Figure 8) substantially compromises its functionality. Interestingly, glutamate (E^117^) residue is highly conserved in the HapR homologues of other *Vibrio* species (Figure 9). It would be interesting to examine the effect of such mutation on the functionality of HapR homologues of different *Vibrio* species. Studies being pursued in our laboratory will possibly shed more insight into the encoded balance between the sequence content, global fold and functionality of HapR homologues, and our current work will be interesting to community involved in shape-function studies of proteins, particularly *Vibrio* species.
